# A Novel Connectome-based Electrophysiological Study of Subjective Cognitive Decline Related to Alzheimer’s Disease by Using Resting-state High-density EEG EGI GES 300

**DOI:** 10.3390/brainsci10060392

**Published:** 2020-06-19

**Authors:** Lazarou Ioulietta, Georgiadis Kostas, Nikolopoulos Spiros, Oikonomou P. Vangelis, Tsolaki Anthoula, Kompatsiaris Ioannis, Tsolaki Magda, Kugiumtzis Dimitris

**Affiliations:** 1 Information Technologies Institute, Centre for Research and Technology Hellas (CERTH-ITI), 57001 Thessaloniki, Greece; kostas.georgiadis@iti.gr (G.K.); nikolopo@iti.gr (N.S.); viknmu@iti.gr (O.V.P.); tsolakianthoula@gmail.com (T.A.); ikom@iti.gr (K.I.); tsolakim1@gmail.com (T.M.); 2 1st Department of Neurology, G.H. “AHEPA”, School of Medicine, Faculty of Health Sciences, Aristotle University of Thessaloniki (AUTH), 54124 Thessaloniki, Greece; 3 Informatics Department, Aristotle University of Thessaloniki (AUTH), 54124 Thessaloniki, Greece; 4 Greek Association of Alzheimer’s Disease and Related Disorders (GAADRD), 54643 Thessaloniki, Greece; 5 Department of Electrical and Computer Engineering, Faculty of Engineering, Aristotle University of Thessaloniki, 54124 Thessaloniki, Greece; dkugiu@auth.gr

**Keywords:** electroencephalography, subjective cognitive decline, brain connectivity, mild cognitive impairment, Alzheimer’s disease, resting state, network analysis

## Abstract

Aim: To investigate for the first time the brain network in the Alzheimer’s disease (AD) spectrum by implementing a high-density electroencephalography (HD-EEG - EGI GES 300) study with 256 channels in order to seek if the brain connectome can be effectively used to distinguish cognitive impairment in preclinical stages. Methods: Twenty participants with AD, 30 with mild cognitive impairment (MCI), 20 with subjective cognitive decline (SCD) and 22 healthy controls (HC) were examined with a detailed neuropsychological battery and 10 min resting state HD-EEG. We extracted correlation matrices by using Pearson correlation coefficients for each subject and constructed weighted undirected networks for calculating clustering coefficient (CC), strength (S) and betweenness centrality (BC) at global (256 electrodes) and local levels (29 parietal electrodes). Results: One-way ANOVA presented a statistically significant difference among the four groups at local level in CC [F (3, 88) = 4.76, *p* = 0.004] and S [F (3, 88) = 4.69, *p* = 0.004]. However, no statistically significant difference was found at a global level. According to the independent sample *t*-test, local CC was higher for HC [M (SD) = 0.79 (0.07)] compared with SCD [M (SD) = 0.72 (0.09)]; t (40) = 2.39, *p* = 0.02, MCI [M (SD) = 0.71 (0.09)]; t (50) = 0.41, *p* = 0.004 and AD [M (SD) = 0.68 (0.11)]; t (40) = 3.62, *p* = 0.001 as well, while BC showed an increase at a local level but a decrease at a global level as the disease progresses. These findings provide evidence that disruptions in brain networks in parietal organization may potentially represent a key factor in the ability to distinguish people at early stages of the AD continuum. Conclusions: The above findings reveal a dynamically disrupted network organization of preclinical stages, showing that SCD exhibits network disorganization with intermediate values between MCI and HC. Additionally, these pieces of evidence provide information on the usefulness of the 256 HD-EEG in network construction.

## 1. Introduction

Alzheimer’s disease (AD) is a neurodegenerative disorder which causes brain pathology and functional changes 10–20 years before the first clinical manifestations [1]. The investigation of the brain connectome in order to find any possible trigger mechanisms in people who will progress to AD, is currently one of the most challengeable research topics in the neuroscience field [2,3,4]. Therefore, the brain connectome could be very promising for shedding light on the potential associations of preclinical stages, such as mild cognitive impairment (MCI) and subjective cognitive decline (SCD), and future cognitive decline related to AD; by searching for similar connectivity disruptions that commonly occur in the AD stage.

### 1.1. Clinical Validity of SCD in AD Continuum

When people age, some of them experience impairment in cognitive functions, without exhibiting clinical manifestations of AD. This particular cognitive status, which is presented before severe dementia, is named MCI [5], whereas the subjective concern of problems related to memory by the elderly without indicative neuropsychological abnormal results is called SCD [6]. Both conditions are linked with wide brain modifications, as well as cognitive decline related to dementia [7,8,9,10,11,12,13]. However, it is unclear which of the SCD individuals will progress to MCI and AD [14,15]. On the other hand, individuals with SCD present similar brain alterations and spatial profiles to more advanced stages of the AD continuum [10,16,17,18,19,20,21,22,23,24,25]. The similarities between these alterations have been verified through the use of brain connectivity networks of people with SCD [25,26,27,28,29] by exploring the brain connectome and network metrics by using graph analysis [17,19,25,30,31,32]. Subsequently, it is imperative to investigate and explore the neurophysiological, mental and cognitive impairment of individuals that should be expected to be in cognitive and psychological statuses compatible with their age and education. In this way, we will be able to acquire better information about AD, its transitional preclinical stages and the associations with changes and disruptions in brain structure and function.

### 1.2. Brain Connectome in SCD

Several neuroimaging studies, using Magnetic Resonance Imaging (MRI) as a neuroimaging tool, have explored multiple network properties of people with multiple brain diseases [33,34] and in early stages of the dementia spectrum (e.g., SCD and MCI) [17,19,25,26,27,28,29,30,31,32,35,36,37,38,39,40,41,42,43,44,45,46,47,48]. In particular, it has been proposed that functional connectivity (FC) changes in people who may probably develop AD after some time might occur before neuropsychological deficits and extensive structural and functional brain interruptions [26,48,49,50,51,52,53] take place. Many studies have reported that people with AD, from the pre-dementia to dementia stages, have significant hub-concentrated lesion distributions [54,55]. Additionally, there are pieces of evidence suggesting that the disruption of FC basically includes areas of the posterior default mode network (DMN). More specifically, the posterior cingulate cortex, which is considered as a “key hub of the DMN” is mostly interrupted in the earliest stages of AD and MCI [42,56,57,58,59], underlying reduced FC among brain areas of the parietal and occipital regions. Moreover, early existing research has found different brain alterations in healthy controls compared to amnestic MCI and AD patients during the encoding process over the superior parietal lobe, cingulate cortex, middle temporal lobe and precuneus [60,61,62]. Despite the fact that a wide variety of the previous reported studies have found that connectivity over multiple brain regions was partially interrupted in SCD compared to healthy controls, a great amount of network properties was widely preserved in the first ones [63], which underlines that the brains of SCD individuals still maintain a few network properties since their brains have not been completely damaged. These findings reveal that the disintegrated strength of the DMN nodes of posterior and temporal brain areas, with a simultaneous increase in nodal strength over anterior regions, is manifested in SCD individuals in a similar way as in the more advanced dementia spectrum (e.g., MCI and AD) [64].

### 1.3. Overview of Sections of the Manuscript

The following section (Section 2) presents the materials and methods of the cross-sectional study on the four groups of people with cognitive impairment within the dementia spectrum, ranging from SCD to AD, compared to HC. The first subsection (Section 2.1) describes the setting of the study and the participants’ characteristics, while the two following subsections (Section 2.2 and Section 2.3) present in detail the neuropsychological assessment of the participants and the electroencephalography (EEG) recording protocol. The fourth subsection (Section 2.4) presents the EEG network analysis, the connectivity measures and network metrics (Section 2.5) we used and presents in detail the EEG acquisition process, while Section 2.5.2 presents the statistical analysis we applied. Furthermore, Section 3 presents a detailed description of the estimation methods and data analysis between the groups (Section 3.1, Section 3.2 and Section 3.4), while the sensitivity and specificity of the network metrics are also reported (Section 3.3). Section 4 underlines the main outcomes of the research and compares them with existing similar approaches, while Section 5 underlines the main conclusion of the manuscript and presents future research questions.

### 1.4. Study Aim

To the best of our knowledge, the present study constitutes novel research focusing on utilizing graph metrics as derived from electrophysiological data from high-density electroencephalography (HD-EEG, EGI GES 300, CERTH-ITI, Thessaloniki, Greece), in order to investigate network differences throughout multiple preclinical stages of the AD continuum, including SCD and MCI, as well as AD compared to healthy control individuals (HC). It has been proven that brain localization using HD-EEG with 256 or 128 arrays is more sensitive, providing sufficient results in brain disorders in contrast with 32-channel array EEG [65], while several existing studies have come to the conclusion that a spacing of less than 2 cm between electrodes can provide insightful information about brain activity [65,66,67,68]. Thus, we were interested in investigating whether network analysis with HD-EEG has clinical and scientific importance as a neuroimaging tool to find any network disruptions among people at preclinical stages of AD. Therefore, we implemented HD-EEG resting state activity and we constructed correlation matrices and weighted undirected networks to precisely detect brain network properties across the AD spectrum and compared the results with standard neuropsychological tests. Electrophysiological metrics generated from neuroimaging tools, such as EEG, have been demonstrated as useful instruments for detecting various pathological conditions affecting brain activity, such as AD [3,35,69,70,71,72,73,74,75,76,77]. In light of previous research findings, in the present study, it was expected to find differences in network properties among SCD individuals compared to HC. In particular, we hypothesized that the SCD group would exhibit brain changes and network interruptions in a similar way to those displayed in MCI, although to a lower extent, yielding an intermediate stage between HC and MCI.

Therefore, we aimed to explore the abovementioned assumption by testing the possible sensitivity of three network metrics: (i) clustering coefficient, (ii) strength and (iii) betweenness centrality at both global (whole-brain level) and local levels (parietal area) in several stages of the AD continuum, including individuals with AD, MCI and SCD compared to HC. To the best of our knowledge, there is no existing study that has explored the potential of these particular network metrics in the EEG resting state activity of SCD populations [78].

## 2. Materials and Methods

### 2.1. Settings and Participants

In total, 112 participants were recruited from the memory and dementia clinic of the Greek Association of Alzheimer’s Disease and Related Disorders (GAADRD) and the 1st Department of Neurology, U.H. AHEPA, Aristotle University of Thessaloniki, Greece. The study was carried out in accordance with the Declaration of Helsinki and received approval by the Scientific and Ethic Committee of GAADRD (No56_27/11/2016), and written informed consent was obtained from all participants prior to their participation in the study. The diagnosis of AD was done by a neuropsychiatrist (MT) according to the medical history, neuropsychological tests, structural magnetic resonance imaging (MRI) and clinical and neurological examinations. Twenty individuals had several head or eye movement artifacts, and hence were excluded from further EEG data analysis, yielding 92 participants for the final inclusion in the study.

In detail, the SCD group consisted of 20 participants (mean ± SD: age = 64.9 ± 7.92), the MCI group consisted of 30 participants (mean ± SD: age = 70.40 ± 5.96), the AD group consisted of 20 participants (mean ± SD: age = 73.20 ± 8.17), while 22 HCs were also included, having a similar range of ages (mean ± SD: age = 67.22 ± 4.03). Each participant from the four groups was over 60 years old [79,80,81]. Table 1 presents the average age with the standard deviation for each group of participants. Participants with AD fulfilled the National Institute of Neurological and Communication Disorders and Stroke/Alzheimer’s Disease and Related Disorders Association (NINCDS-ADRDA) criteria for probable AD [82], as well as the Diagnostic and Statistical Manual of Mental Disorders (DSM-V) criteria for dementia of Alzheimer’s type (American Psychological Association, 1994). On the other hand, the MCI participants fulfilled the Petersen criteria [83], while the SCD group met International Working Group -2 guidelines [84] and the recent National Institute on Aging-Alzheimer’s Association workgroups on diagnostic guidelines for Alzheimer’s disease (NI-AA) [85], as well as the SCD-I Working Group instructions [86]. Regarding the preclinical stage of SCD, we tried to eliminate possible confounding factors based on blood tests (hormonal disorders, vitamin deficiency, etc.), structural MRI (vascular/demyelinating lesions, tumors, anatomical variations, etc.) and the qualitative evaluation of the resting state EEG. All the above were taken under consideration for the group recruitment process, as they could have affected our sample performance and our signal elicitation. The criteria for recruiting SCD participants were in accordance with the latest suggestions proposed by the SCD-I Working Group [86]. Moreover, we additionally strived to exclude participants where other etiologies could explain self-perceived memory deficits, including vascular (examination of ischemic lesions of MRI, blood testing), psychiatric (interview, depression scale, psychoactive drugs, etc.) or other systematic etiologies, by carefully evaluating laboratory results, including blood samples, structural MRI, the patient’s medical history and additional questionnaires following the SCD-I Working Group criteria.

The identification of SCD participants further included a set of criteria, which were administered in our previous study [10], as well in other similar approaches [11,23,87] including: “self-perceived memory decline compared to other cognitive functions, and in reference to others of the same age, occurring during the past five years as determined by the individual’s medical history and psychological report, at an age cut-off of 60”. Additional inclusion criteria for the SCD and HC subjects were to have a normal general medical, neurological and neuropsychological examinations. Exclusion criteria included: (i) severe physical, psychiatric or other neurological disorder illness or any other somatic disorder which may cause cognitive impairment, (ii) history of drug or alcohol consumption and the use of neuro-modifying drugs, except cholinesterase inhibitors or memantine for AD and (iv) left handedness.

### 2.2. Neuropsychological Assessment

All participants underwent a detailed neuropsychological assessment, which included a standardized neuropsychological examination, an insightful psychological interview using the Structured Clinical Interview for DSM-IV Axis I Disorders Clinical Version (SCID-CV) [88] and a medical history, as well as physical and neurological examinations. In particular, the following neuropsychological batteries were implemented in order to comprehensively evaluate working memory, executive functioning, attention and memory and language to assess cognitive status: (a) Global Deterioration Scale (GDS) [89], (b) Brief Cognitive Rating Scale (BCRS) [90], (c) the Greek version of the Mini Mental State Examination (MMSE) [91], (d) Rey–Osterrieth Complex Figure Test copy and delay recall (ROCFT copy and delayed recall) [92], (e) Rivermead Behavioral Memory Test (RBMT) story direct and delayed recall [93], (f) Rey Auditory Verbal Learning Test (RAVLT), (g) F.A.S [94], (h) Trail Making Test part B [95], (i) Functional Rating Scale for Dementia (FRSSD) and (j) Functional and Cognitive Assessment Test (FUCAS) [96]. The evaluation of mood and behaviour was carried out using both the interview data and the participants’ answers to the relative brief self-report tools, the Neuropsychiatric Inventory (NPI) [97] and the Perceived Stress Scale (PSS) [98].

### 2.3. Resting State EEG Recording

Fifteen-minute resting EEG activity was recorded for all the participants. For the whole duration of the resting state EEG recording, participants were advised to keep themselves relaxed as much as possible, close their eyes and open them after the researcher’s demand, sit still, minimize blinking or mouth movements and let their mind wander. The experimental procedure was monitored by a research assistant aiming to identify cases of horizontal eye movements, continued blinking or excessive movement by visually inspecting the EEG traces during the experiment. More specifically, an EEG was registered for both resting conditions (eyes open, EO and eyes closed, EC) for at least 2–3 min for each period.

### 2.4. EEG Data Acquisition and Network Construction

**Figure 1:**
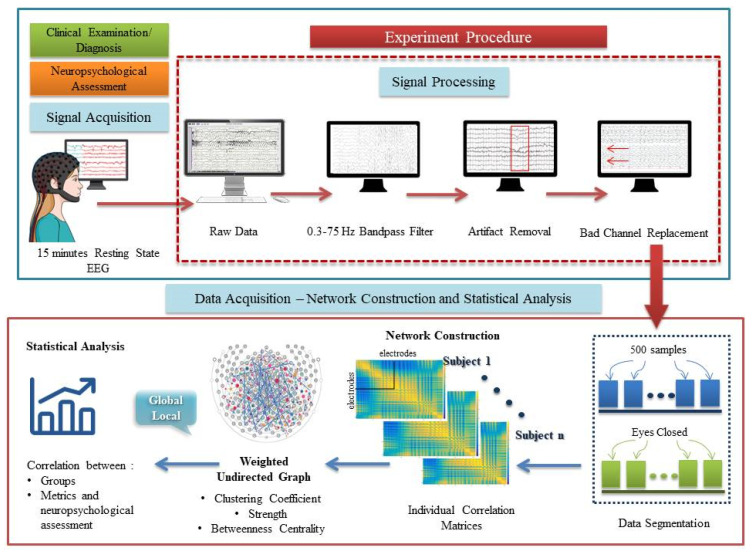
Outline of the methodology for extracting the network metrics derived from correlation matrices.

We followed the same protocol as we did in our previous research efforts [10]. In particular, the EEG data were collected by using the EGI 300 Geodesic EEG system (GES 300, CERTH-ITI, Thessaloniki, Greece) with a 256-channel HydroCel Geodesic Sensor Net (HCGSN) and a sampling rate of 250 Hz (EGI Eugene, OR). Moreover, the researcher placed the electrodes in accordance with the 256 HCGSN adult 1.0 montage system, while the signals were recorded relative to a vertex reference electrode (Cz), with AFz as the ground electrode with the electrodes’ impedance below 50 kΩ throughout the experimental procedure, as recommended [99] for the high-input impedance amplifier. In detail, the HD-EEG data were analyzed offline in order to detect any artifact, as well as to conduct pre-processing (filtering, segmentation, bad channel replacement) using Net Station 4.3 software (EGI). Figure 1 illustrates the pipeline process for data acquisition, the construction of the weighted undirected networks and the extraction of the metrics derived from the correlation matrices of the resting state EEG.

Moreover, HD-EEG data were initially filtered with 5th-order bandpass Butterworth IIR filter of 0.3–75 Hz and then segmented using a 500-sample non-overlapping window. We examined only the eyes closed period. Once the segmentation was completed, the detection of artefacts was performed by using the Net Station artefact detection tool for the automatic detection of excessive eye blinking and movement. The detection of “bad” segments was executed by marking those segments with amplitudes more than 100 µV. Additionally, signals from the rejected (bad) electrodes were replaced using an interpolation process provided by the “bad channel replacement” algorithm (Net Station 4.3). Afterwards, the signals were baseline corrected using 200 msec before the start of the experiment period and average re-referenced to transform them into reference-independent values. The brain network analysis was conducted at first in a personalized fashion, deriving the individual weighted correlation matrices (absolute values) over broadband activity upon all trials. Then the averaged profiles (i.e., group-averaged to demonstrate them for comparison purposes) were estimated for every group (HC, SCD, MCI and AD) and were considered as the input matrices (static brain networks) for the estimation of the network metrics (strength, clustering coefficient and betweenness centrality) from fully weighted networks. Besides the pre-processing steps performed using Net Station’s algorithms, all other processing and analysis steps were performed using Matlab 2018b (The Mathworks, Natick, MA, USA).

### 2.5. Connectivity: Pearson Correlation Coefficient (PCC)

PCC was implemented in order to measure connectivity between all pairs of electrodes. PCC is a measure of normalized covariance between two continuous variables that can be estimated by dividing the covariance of two variables by the product of their standard deviations, given as

$$r_{XY}=\frac{\sum_{i=1}^{n}\left(X_{i}-\bar{X}\right)\left(Y_{i}-\bar{Y}\right)}{\sqrt{\sum_{i=1}^{n}{\left(X_{i}-\bar{X}\right)}^{2}}\sqrt{\sum_{i=1}^{n}{\left(Y_{i}-\bar{Y}\right)}^{2}}}$$

where *X* and *Y* are two channels and the corresponding EEG measurements in a segment and $\bar{X}$ is their mean. Weighted matrices were created using the PCC between the time series of each pair of electrodes (all electrodes at a global level, only selected parietal electrodes at a local level). The absolute values of the PCC were used in order to estimate the respective network metrics.

#### 2.5.1. Global Brain and Local Parietal Network Analysis

Correlation matrices were constructed from the EEG measurements and used as weighted adjacency matrices. Network characteristics were derived from the weighted adjacency matrices, including clustering coefficient, network strength and betweenness centrality, to characterize the connectivity properties of global brain and local parietal network using the Brain Connectivity Toolbox and FieldTrip Toolbox. In detail, we considered a local network of selected electrodes (parietal region), which is the most prominent choice for the examination of resting state network(s) [35,73,75,100,101,102]. Regarding the local parietal network, we chose the following electrodes according to the EGI system, numbering: 78, 87, 100, 101, 63, 142, 154, 35, 87, 99, 110, 119, 63, 141, 153, 163, 86, 98, 109, 118, 127, 140, 152, 162, 96, 97, 108, 170 and 161 [103], which represent the respective parietal area.

In the present study, we implemented graph analysis so as to seek for any significant differences among the four groups (HC, SCD, MCI and AD) with regards to brain connectivity. The corresponding channels of the EEG constitute the nodes of the graph, while the correlations between the node electrodes (absolute value of PCC) represent the edges of the graph. We constructed a weighted graph in order to analyze the brain network and explore the network metrics we chose (clustering coefficient, strength and betweenness centrality). While strength (S) quantifies aggregation and clustering coefficient (CC) segregation, we also considered the betweenness centrality (BC) measure as a measure of centrality [104]. The three metrics are briefly presented below.

##### Clustering Coefficient (CC)

Given a graph *G* of *N* nodes and weighted connections, the weighted clustering coefficient $C_{i}^{w}$ of node *i* provides us with a measure of interconnection between node *i* and its neighbors [104]. The overall weighted clustering coefficient $C^{w}$ of the graph *G* is computed as the average of $C_{i}^{w}$ over all nodes *i*:

$$C^{w}=\frac{1}{N}\overset{N}{\underset{i=1}{\sum }}C_{i}^{w}$$

##### Strength (S)

Next, the connection strength $S_{i}$ of each node *i* in the graph is estimated as the sum of the weights of all the connections of node *i*, gaining information on the total level of the (weighted) connectivity of a node [104]. The strength expresses how strongly the node is connected with its neighboring nodes, by summing all weights of the connections of this node. For a weighted undirected graph, the strength of node *i* is simply the sum of the components in the *i*-th row or column of the weight matrix. The total strength *S* of the graph *G* is the average of all *N* node strengths:

$$S=\frac{1}{N}\overset{N}{\underset{i=1}{\sum }}S_{i}$$

##### Betweenness Centrality (BC)

Finally, the betweenness centrality *BC_v_* of a node *v* in the graph is related to the fraction of the total number of shortest paths that pass through node *v* from node *i* to node *j* (*σ_ij_*(*v*)) to the total number of shortest paths from node *i* to node *j* (*σ_ij_*) [104]. BC describes the centrality of a graph using the shortest paths and represents the degree to which nodes stand between each other. The total BC of the graph *G* is the average of all *N* node betweenness centralities:

$$BC=\frac{1}{N}\overset{N}{\underset{i=1}{\sum }}BC_{i}$$

#### 2.5.2. Statistical Analysis

We compared brain network data (in terms of PCC) among the four groups at the level of significance *p* = 0.05. The network metrics of the global and local networks were compared between groups using ANOVA analysis. Exploratory correlation analysis tested the relationship of global and local network metrics with neuropsychological test scores of participants using the PCC (*p* = 0.05, uncorrected for multiple comparisons) so as to explore the potential connection between cognitive performance and how this is interpreted in network metrics.

Statistical analysis was performed using SPSS v25.0 for Windows (IBM Corporation, Armonk, NY, USA) and R Studio software. For assessing the normality assumption for continuous and categorical variables, we used the Kolmogorov–Smirnov and chi-squared test, respectively. For examining the potential statistical significance between two independent groups (e.g., HC vs. SC) we used the independent sample *t*-test. Moreover, the independent sample *t*-test was used for the years of education variable, yielding no statistical difference among the groups, with *p* = 0.253, while no gender differences were found with respect to gender after chi-squared analysis (*p* = 0.522). Despite that, in each group of participants, the female participants were more in total compared to male participants, a finding indicative of the prevalence of AD [105,106,107]. However, with respect to age, although we included participants over 60 years old, a statistically significant difference was found in the AD group compared to HC (*p* = 0.01). Nevertheless, no statistically significant difference was found either among HC and the preclinical groups of SCD (*p* = 0.127) and MCI (*p* = 0.09) or between SCD and MCI (*p* = 0.690). The independent sample *t*-test was also used in order to find any potential statistically significant difference in neuropsychological tests among groups. We used one-way ANOVA in order to analyze the difference in the network metrics across the four groups. In cases where graph measures showed statistical significance between groups, within group differences were tested using the *t*-test for independent samples (*p*-values were reported and interpreted in view of the Bonferroni correction for multiple comparisons on the statistical significance level). Correlation between neuropsychological tests and network-derived metrics was assessed by using Pearson correlation coefficient.

## 3. Results

### 3.1. Neuropsychological Assessment of HC, SCD, MCI and AD

In each of the neuropsychological tests we included to test the cognitive performance of the four groups, the performance of all HC and SCD participants was indicative of normal cognitive status (Table 2). Nevertheless, according to the one-way ANOVA test, the MCI and AD groups showed statistically significantly worse performance scores in the majority of MMSE subsections, FRSSD and FUCAS items, RAVLT, FAS, ROCFT and RBMT memory tests. Superscripts show the statistical significance among the four groups after independent sample *t*-tests.

In detail, the one-way ANOVA between subjects was conducted to compare the cognitive status and behavioral issues that commonly arise in the AD spectrum by administering standardized neuropsychological tests and define the limitations in each group. There was a significant effect of diagnosis on several neuropsychological tests at the *p* < 0.05 level among the four groups, as follows: MMSE: [F(3, 88) = 42.35, *p* = 0.0001], FRSSD total score: [F(3, 88) = 5.55, *p* = 0.002], FUCAS total score: [F(3, 88) = 9.76, *p* = 0.0001], TRAIL-B: [F(3, 88) = 5.54, *p* = 0.002], RBMT immediate recall: [F(3, 88) = 3.89, *p* = 0.015], ROCFT delayed recall: [F(3, 88) = 11.71, *p* = 0.0001], RAVLT immediate recall: [F(3, 88) = 3.14, *p* = 0.035] and RAVLT total score: [F(3, 88) = 7.07, *p* = 0.001]. In order to further investigate the differences among each pair of groups, we conducted independent sample *t*-tests, which indicated the following results.

**Global Cognition**: According to independent sample *t*-tests, the MMSE score was better for HC (M = 29.13, SD = 0.99) compared with MCI (M = 27.13, SD = 2.55), t (50) = 3.48, *p* = 0.001, and AD groups (M = 22.30, SD = 3.35), t (40) = 9.13, *p* < 0.0001. In this common vein, the SCD (M = 29.25, SD = 1.06) group also outperformed MCI (M = 27.13, SD = 2.55), t (48) = 3.49, *p* = 0.001, and AD t (38) = 8.82, *p* < 0.0001, and in turn MCI outperformed AD, t (48) = 5.77, *p* < 0.0001. The differences were also found to be statistically significant under the Bonferroni correction for multiple testing, which here was equivalent to setting the significance level to 0.008.

**Daily Functionality**: Independent sample *t*-tests revealed that the FRSSD total score was better for HC (M = 1.58, SD = 2.50) compared to MCI (M = 4, SD = 1.51), t (32) = −3.52, *p* = 0.001 and AD groups (M = 6.75, SD = 6.60), t (40) = −2.28, *p* = 0.03. There was also a significant difference in the scores for FRSSD total score between HC (M = 1.58, SD = 2.50) and SCD (M = 3.20, SD = 1.57), t (30) = −2.25, *p* = 0.032, but both were within normal range. For the FUCAS test, the HC group (M = 42.0, SD = 0.00) outperformed both MCI (M = 44.77, SD = 3.41), t (32) = −2.79, *p* = 0.009 and AD (M = 50.37, SD = 8.99), t (38) = −3.27, *p* = 0.004. Moreover, the SCD group (M = 42.55, SD = 1.27) demonstrated better performance than MCI t (40) = −2.75, *p* = 0.009 and AD t (38) = −3.90, *p* = 0.001. Finally, the MCI group had greater scores than AD in the FRSSD total score t (48) = −1.87, *p* = 0.07 and FUCAS total score t (48) = −2.52, *p* = 0.01. The differences were also found to be statistically significant between HC and MCI under the Bonferroni correction for multiple testing for daily functionality measurements (except the subcategory of FUCAS memory and FRSSD personal hygiene), which here was equivalent to setting the significance level to 0.008.

**Memory and Executive Function**: HC (M = 143, SD = 54.86) had better scores than MCI (M = 262.42, SD = 137.61), t (50) = −2.86, p = 0.007 in TRAIL part B. Additionally, independent sample t-tests revealed that the RBMT immediate recall was better for HC (M = 17.4, SD = 2.70) compared to MCI (M = 12.71, SD = 4.04), t (50) = 2.44, p = 0.022 and AD (M = 10.30, SD = 2.49), t (40) = 4.32, p = 0.003. Additionally, HC showed better performance compared to AD (M = 9.50, SD = 3.31), t (40) = 3.37, p = 0.01 in RBMT delayed recall, as well. Additionally, HC (M = 31, SD = 1.41) had better performance than the MCI group (M = 13.54, SD = 5.76), t (50) = 4.18, p = 0.0001 and AD (M = 9.90, SD = 9.16), t (40) = 3.06, p = 0.02 in ROCFT delayed recall. In this common vein, SCD (M = 144.75, SD = 49.64) had better scores than MCI (M = 262.42, SD = 137.61), t (35) = −3.25, p = 0.003 in TRAIL part B. Moreover, SCD (M = 22.08, SD = 5.69) had better performance than the MCI group (M = 13.54, SD = 5.76), t (35) = 4.48, p = 0.0001 and AD group (M = 9.90, SD = 9.16), t (38) = 3.61, p = 0.002 in ROCFT delayed recall. Moreover, SCD had better performance in RBMT - immediate recall (M = 14.18, SD = 3.28) and RBMT delayed recall (M = 13.09, SD = 3.23), as well as ROCFT copy (M = 33.68, SD = 1.64) compared to the AD group [RBMT—immediate recall: (M = 10.30, SD = 2.49), t (38) = 2.42, p = 0.02, RBMT delayed recall: (M = 9.50, SD = 3.32), t (38) = 2.16, p = 0.04 and ROCFT copy: (M = 22.80, SD = 13.43), t (38) = 3.35, p = 0.003]. Finally, the MCI group (M = 30.23, SD = 5.05) also demonstrated significantly better performance with respect to the AD group (M = 22.80, SD = 13.43), t (48) = 2.08, p = 0.004 in ROCFT copy test. The differences were also found to be statistically significant under the Bonferroni correction for multiple testing in the majority of memory and executive function neuropsychological tests, which here was equivalent to setting the significance level to 0.008. However, no statistically significant differences were found between HC and MCI in TRAIL part B and RBMT delayed recall, and SCD and MCI were similar in ROCFT delayed recall, where the Bonferroni correction for multiple testing was not equivalent to setting the significance level to 0.008.

**Verbal Fluency—Learning**: HC (M = 53.33, SD = 13.86) outperformed MCI (M = 33.38, SD = 16.09), t (31) = 2.09, *p* = 0.05 in RAVLT total score. tThe independent sample *t*-test revealed that the FAS total score was better for HC (M = 14.3, SD = 3.20) compared to MCI (M = 9.49, SD = 3.75), t (31) = 2.09, *p* = 0.04. Moreover, SCD (M = 53.33, SD = 13.86) outperformed MCI (M = 33.38, SD = 16.09), t (36) = 4.29, *p* = 0.0001 in RAVLT total score. In addition to that, SCD showed better performance in RAVLT-2 (M = 7.35, SD = 3.83) and RAVLT immediate recall (M = 7.23, SD = 2.75) compared to MCI (M = 5.33, SD = 2.19), t (36) = 2.03, *p* = 0.04 and (M = 5.19, SD = 2.08), t (36) = 2.60, *p* = 0.01, respectively. Moreover, SCD showed significantly better performance in RAVLT total score (M = 53.88, SD = 12.56) compared to AD (M = 34.0, SD = 16.85), t (38) = 2.88, *p* = 0.009. The independent sample *t*-test revealed that the FAS total score was better for SCD (M = 12.18, SD = 3.20) compared to MCI (M = 9.49, SD = 3.75), t (36) = 2.19, *p* = 0.03. The differences were also found to be statistically significant under the Bonferroni correction for multiple testing in memory and executive function neuropsychological tests, which here was equivalent to setting the significance level to 0.008.

**Mood**: Lower scores, which indicate better performance, for HC (M = 0.00, SD = 0.00) and SCD (M = 0.33, SD = 0.73) were found in NPI compared with the AD group (M = 2.75, SD = 4.23), t (40) = −2.28, *p* = 0.03 and (M = 2.75, SD = 4.23), t (38) = −2.56, *p* = 0.01, respectively. The differences were also found to be statistically significant under the Bonferroni correction for multiple testing in mood assessment tests, which here was equivalent to setting the significance level to 0.008. Nevertheless, no group had clinical manifestations of depression or anxiety disorder since the mean scores for NPI and PSS were below the cut-off scores.

Consequently, statistically significant differences were found in many neuropsychological tests among the four groups (HC, SCD, MCI and AD), supporting the differentiation of MCI and AD compared to HC in a variety of cognitive domains (e.g., daily functionality, memory, executive function, etc.). However, as expected, in the case of SCD and HC, no significant differentiation was found between traditional neuropsychological tests, which paves the way to explore other mechanisms to detect SCD. Thus, taking into account the absence of any differentiation between HC and SCD, we explored the likelihood of any potential difference between the four groups, as well as between HC and SCD, with regards to the brain connectome in a resting state condition.

### 3.2. Comparison of Network Properties between HC, SCD, MCI and AD

**Figure 2:**
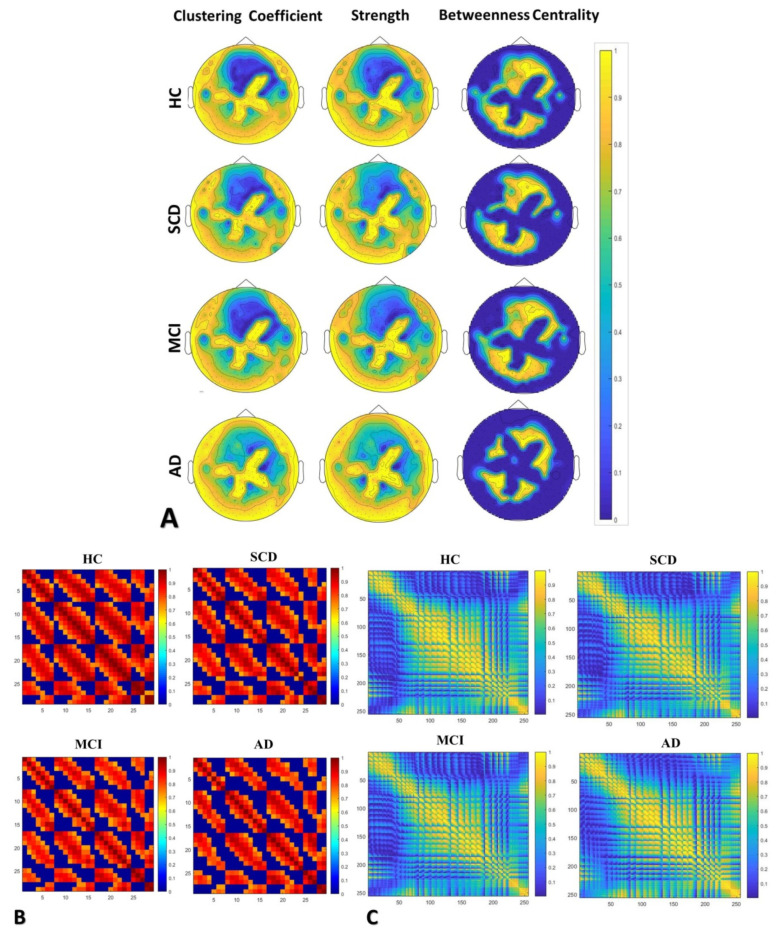
(**A**) Topo plots for the three network metrics (clustering coefficient, strength and betweenness centrality), (**B**) correlation matrices of local networks (29 parietal electrodes) and (**C**) correlation matrices of global networks (256 electrodes) across the four groups of participants (HC = 22, SCD = 20, MCI = 30, AD = 20).

**Figure 3:**
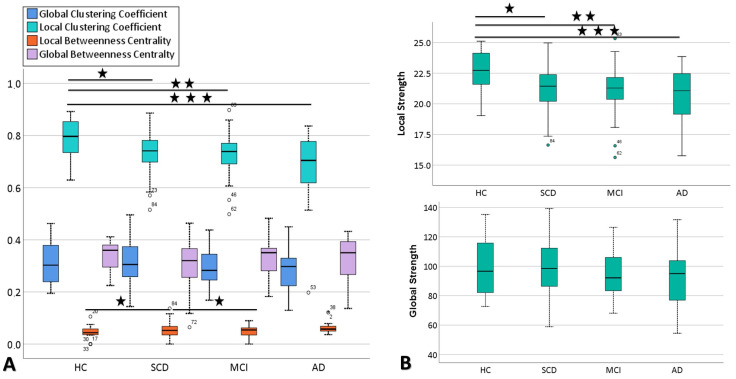
Boxplots showing differences in global and local network metrics for the separate groups of participants (HC = 22, SCD = 20, MCI = 30, AD = 20) in (**A**) clustering coefficient and betweenness centrality and (**B**) strength.

As presented in Table 3 alongside the network properties measured (clustering coefficient, strength and betweenness centrality), mean values of the HC group were higher compared to all other groups (i.e., SCD, MCI and AD). Superscripts indicate statistically significant differences between the groups after the independent sample *t*-test was performed. Moreover, Figure 2B,C illustrate the correlation matrices at local and global levels, respectively, from which the network was constructed in order to estimate the network metrics and create the topoplots presented in Figure 2A. From the matrices of global and local networks, we constructed weighted undirected networks for each group of participants, as shown in Figure 2B,C, respectively. Moreover, the mean values and SD of clustering coefficient and betweenness centrality, as derived from local and global networks, are illustrated in Figure 3A, while the local and global strength mean and SD values are depicted in Figure 3B. A one-way ANOVA was conducted to compare each group of participants in each network property at local and global levels. There was a significant effect of diagnosis on every network property at a local level (parietal electrodes) at a 0.05 level among the four groups in clustering coefficient: [F (3, 88) = 4.76, *p* = 0.004], strength: [F (3, 88) = 4.69, *p* = 0.004] and betweenness centrality: [F (3, 88) = 3.50, *p* = 0.681]. However, no statistically significant difference was found at a global level between the four groups in global clustering coefficient: [F (3, 86) = 0.50, *p* = 0.681], global strength: [F (3, 88) = 0.67, *p* = 0.569] and betweenness centrality: [F (3, 88) = 0.48, *p* = 0.53]. Independent sample *t*-tests indicated that significant differences were found in network metrics, as presented below, and especially at a local level.

**Global and Local Clustering Coefficient (CC):** According to independent sample *t*-tests, the local clustering coefficient was higher for HC (M = 0.79, SD = 0.07) compared to SCD (M = 0.72, SD = 0.09), t (40) = 2.39, *p* = 0.02, MCI (M = 0.71, SD = 0.09), t (50) = 0.41, *p* = 0.004 and AD groups (M = 0.68, SD = 0.11), t (40) = 3.62, *p* = 0.001. On the other hand, with regard to the global clustering coefficient, comparisons between SCD and MCI, SCD and AD and MCI versus AD revealed no statistically significant differences (Table 3). Despite that, HC (M = 0.31, SD = 0.07) showed greater values with regard to the global clustering coefficient, compared to SCD (M = 0.30, SD = 0.08), t (40) = 0.13, *p* = 0.897, MCI (M = 0.29, SD = 0.07), t (48) = 0.94, *p* = 0.351 and AD (M = 0.28, SD = 0.09), t (40) = 0.97, *p* = 0.337, where no statistically significant difference was found (Figure 3).

**Global and Local Strength (S):** According to independent sample *t*-tests, the local strength at parietal electrodes showed higher values for HC (M = 22.56, SD = 1.65) compared to SCD (M = 21.11, SD = 2.10), t (40) = 2.50, *p* = 0.01, MCI (M = 20.83, SD = 2.25), t (50) = 3.01, *p* = 0.004 and AD groups (M = 20.12, SD = 2.66), t (40) = 3.48, *p* = 0.001. On the other hand, with regard to the global strength, comparisons between SCD and MCI, SCD and AD and MCI versus AD revealed no statistically significant differences (Table 3). Although HC (M = 99.24, SD = 18.08) showed greater values with regard to global strength compared to SCD (M = 97.70, SD = 20.18), t (40) = 0.26, *p* = 0.795, MCI (M = 94.01, SD = 16.20), t (48) = 1.07, *p* = 0.287 and AD (M = 91.88, SD = 21.91), t (40) = 1.18, *p* = 0.245, no statistically significant differences were found between SCD vs. MCI and AD or MCI vs. AD (Figure 3).

**Global and Local Betweenness Centrality (BC):** Based on the independent sample *t*-tests, local BC at parietal electrodes showed statistically significantly lower values for HC (M = 0.04, SD = 0.03) compared to SCD (M = 0.056, SD = 0.03), t (40) = −1.42, *p* = 0.05. Moreover, local BC at parietal electrodes showed statistically significant lower values for MCI (M = 0.04, SD = 0.02) compared to SCD (M = 0.056, SD = 0.03), t (40) = −1.42, *p* = 0.04 (Table 3). On the other hand, with regard to local BC, comparisons between the remainder of the groups revealed no statistically significant differences. Albeit, with regard to global betweenness centrality, all groups showed similar values, while no statistically significant differences were found between SCD vs. MCI and AD or MCI vs. AD (Figure 3).

To sum up, as illustrated in Figure 2A, HC presents a denser network with several connections between nodes in a local area (parietal electrodes) as well as in a global network with regard to CC and strength. As the disease progresses, we can see fewer connections between nodes. Similarly, correlation matrices, as shown in Figure 2B,C for local and global networks, respectively, support the assumption of less connectivity between nodes (electrodes) in later stages. The correlation differences are more obvious in the local network. Especially in the case of AD, the connections of network connections are much more aberrant, while network interruption is widely observed over the global network. Additionally, boxplots in Figure 3 show increased functional connectivity (lower mean values in CC and higher mean strength, Figure 3A) in HC with regards to SCD, MCI and AD participants, while higher mean values of betweenness centrality were found in later stages of the disease.

### 3.3. Sensitivity and Specificity of Network Properties

In the present section, we investigate the potential utility of the abovementioned network properties as markers of an individual’s condition (SCD, MCI and AD) or an HC by testing sensitivity and specificity among the groups. More specifically, we examined the area under the curve (AUC), the sensitivity and specificity. These pieces of evidence can provide information about the use of local or global clustering coefficients, betweenness centrality and strength as tools that would indicate the condition of an SCD individual.

**Figure 4:**
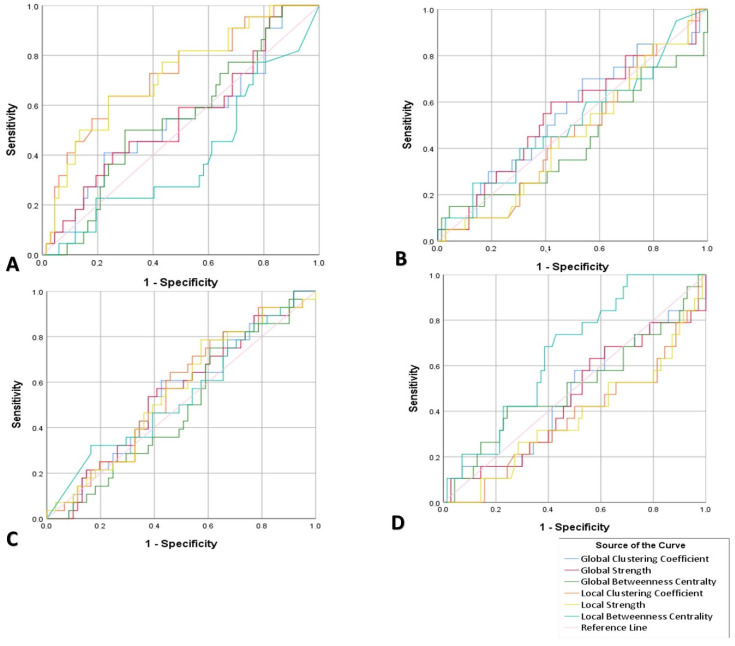
ROC curves presenting for the clustering coefficient, strength and betweenness centrality at local and global levels for discriminating between: (**A**) HC vs. SCD, MCI and AD, (**B**) SCD vs. HC, MCI and AD, (**C**) MCI vs. HC, SCD and AD and (**D**) AD vs. SCD, MCI and HC.

**Figure 5:**
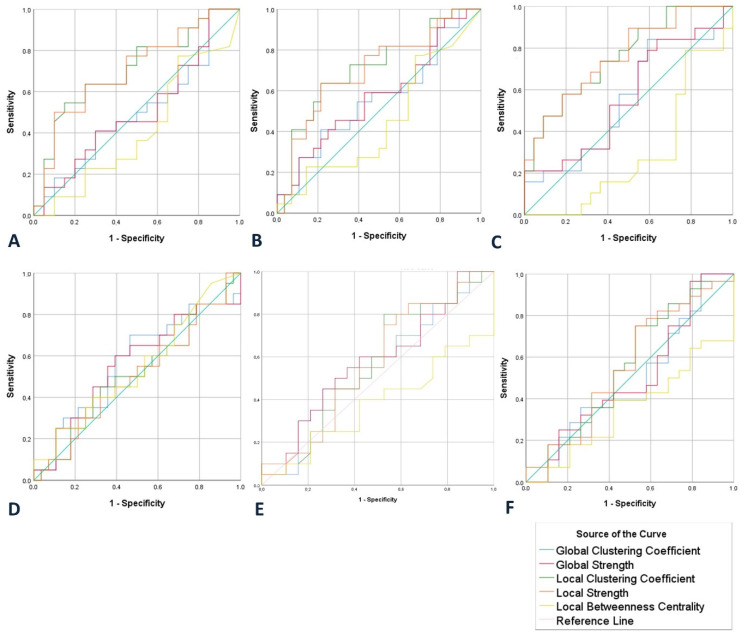
ROC curves showing the clustering coefficient, strength and betweenness centrality at local and global levels for discriminating between: (**A**) SCD and HC, (**B**) MCI and HC, (**C**) AD and HC, (**D**) MCI and SCD, (**E**) SCD and AD and (**F**) MCI and AD.

Specificity and sensitivity values were estimated by using SPSS v25.0. In particular, we developed Receiver operating characteristic curves (ROC)and identified the best threshold of the local and global clustering coefficients, betweenness centrality and strength values to differentiate the groups. Taking into account recent neurophysiological studies, a minimum value of 65% for both sensitivity and specificity constitutes an acceptable rate [10, 108]. The sensitivity and specificity scores corresponding to the cut-off thresholds, alongside the AUC, are shown in Table 4 and Table 5, while Figure 4 and Figure 5 present in detail the results of the AUC, sensitivity and specificity in global and local networks, respectively.

**One vs. Other Groups**

In the one vs. other groups simulation scenario, we only managed to successfully discriminate HC from SCD, MCI and AD using either the clustering coefficient (sensitivity = 64% and specificity = 78%, AUC = 74%) or the strength (sensitivity = 64% and specificity = 79%, AUC = 74%) measures at a local level. None of the other simulation tests managed to yield a performance over the minimum value (AUC = 65%) for both specificity and sensitivity (Figure 4).

**One vs. One**

In the one vs. one simulation scenario, we only managed to successfully discriminate HC from SCD using either the clustering coefficient (sensitivity = 75% and specificity = 64%, AUC = 71%) or the strength (sensitivity = 75% and specificity = 64%, AUC = 71%) measures at a local level. Additionally, we managed to discriminate HC from MCI using either the clustering coefficient (sensitivity = 64% and specificity = 80%, AUC = 73%) or the strength (sensitivity = 80% and specificity = 64%, AUC = 79%) measures at a local level. Finally, HCs were also discriminated from AD using either the clustering coefficient (sensitivity = 65% and specificity = 82%, AUC = 79%) or the strength (sensitivity = 65% and specificity = 82%, AUC = 79%) measures at a local level. None of the other simulation tests managed to yield a performance over the minimum value (AUC = 65%) for both specificity and sensitivity (Figure 5). Consequently, although these results are very promising, there is still work to do to reaching an acceptable level for discriminating each pair of groups.

### 3.4. Correlation between Neuropsychological Assessment and Network Properties

Furthermore, we used a Pearson correlation in order to seek for any potential correlations among the neuropsychological tests and the local network metrics (Table 6). More specifically, we can see that values of sleep, as measured in the FRSSD test, were negatively correlated with the local clustering coefficient at parietal electrodes generated during the resting state EEG. The local clustering coefficient captures how strongly particular nodes are connected with their neighboring nodes, corresponding to specific areas of the brain, showing that the larger the value of CC is in a brain region, the more it affects its neighboring areas of the brain. The local clustering coefficient was found to be negatively correlated with the FRSSD sleep score (r = −0.286, *p* = 0.034) with statistical significance. Moreover, BC was negatively correlated with MMSE (r = -0.254, *p* = 0.04), RBMT delayed recall (r = −0.362, *p* = 0.032) and ROCFT copy (r = −0.501, *p* = 0.025), whereas a statistically significant positive correlation was found between BC and FUCAS total score (r = 0.281, *p* = 0.038) and the FRSSD sleep parameter (r = 0.522, *p* = 0.033), indicating that higher cognitive impairment, as daily functionality problems show, increased BC in the parietal area. Therefore, standardized neuropsychological tests show that, in the case of BC, several cognitive domains (global cognition, episodic memory, visuospatial long-term memory and daily functionality) have a weak but statistically significant correlation, supporting that network disruption and the loss of connection between brain areas may impact cognition, as measured in the neuropsychological assessments.

## 4. Discussion

The present study presents pieces of evidence from investigating brain connectome changes in the preclinical stage of AD by using a resting state HD-EEG, while highlighting the importance of network metrics to find ways for the early detection of SCD and its connectivity mechanisms as a preclinical stage of the AD continuum. Our study confirms and underlines the presence of an interrupted brain connectome in SCD and describes the potential of the brain connectome in order to detect future cognitive decline related to AD. Additionally, it suggests that disordered brain function, characterized by decreased coherence in specific nodes of the brain network, may be related to SCD. This implies that SCD is considered as an intermediate condition between the two stages, healthy ageing and MCI. Since this is the first ever reported study which explored these particular brain network metrics in people with SCD by using HD-EEG, we compare our results with other approaches found in the literature that deployed different modalities (e.g., Magnetoencephalography - MEG, fMRI) or with EEG studies that explored potential differences between HCs and people in more severe stages (e.g., AD or MCI).

Despite the fact that the a wide variety of the resting state studies showed disrupted patterns and interrupted links in SCD compared to HCs [28,30,37,42], there were some with opposing results, presenting increased FC in SCD with regards to HCs [26,32]. More specifically, decreased FC was basically presented among posterior brain regions in SCD compared to the HC group [37,42]. Similarly, in our study, SCD exhibited decreased strength and clustering coefficients in the parietal area compared to HCs. Similar results can also be found in similar brain network studies, demonstrating decreased nodal strength in SCD individuals in key regions of resting state networks basically located over the parietal region [26,32,38,57,109,110], which can be partially explained due to the low levels of glucose in the inferior parietal lobe [111]. Therefore, since the parietal region is the primary target of functional decrease in SCD individuals, which may further lead to cognitive decline associated with more advanced stages of the AD continuum, we found connectivity abnormalities and statistically significant differences basically located over the parietal region as well. Moreover, lower strength and clustering coefficients compared to HC was also found in MCI and AD, both at a global level and in the local parietal network. This implies that all the connections which are interrupted in SCD are also affected in a similar way in MCI, which supports the assumption that both conditions show a common “functional coupling pattern”. These findings suggest that SCD presents intermediate connectivity disruption over posterior regions with regards to HCs and MCI [28,42]. Our results pave the way to imply that the subjective feeling of memory loss without any objective clue of cognitive decline, as revealed from neuropsychological tests, could be indicative of pathological brain function related to future progression to AD.

Similar approaches have demonstrated that HC have increased FC compared to SCD, while a disconnection over posterior regions was observed in SCD with an anterior hyper-synchronization of the exact same brain areas as MCI [29,37,38,45,47] was found. On the other hand, with regards to the nodal clustering coefficient changes, it is worth mentioning that differences were found only between MCI and HC, whereas SCD presented no differences compared to HC or MCI [25]. In contrast to the abovementioned findings, our study found statistically significant differences between SCD and HC with respect to local clustering coefficient. However, we did not find any statistically significant difference between HC and SCD with regard to the global clustering coefficient, as well as between the rest of the groups (SCD vs. MCI, SCD vs. AD and MCI vs. AD). On the other hand, SCD also demonstrated decreased strength, especially between rich-club regions compared to HC [29], proposing a common disconnection pattern of the brain connectome in SCD but milder than in MCI. In this common vein, our study showed that strength values were significantly lower in SCD, AD and MCI compared to HC at a local level but not at a global level, suggesting that by constructing brain networks from resting state EEGs, we can observe intermediate values of the SCD between HC and MCI.

It has been suggested that there is wide disconnection between frontal and parietal brain areas in prodromal AD, supporting an interruption of anterior–posterior connection [19]. Since the posterior cingulate cortex (PCC), inferior parietal lobule (IPL) and retrosplenial cortex (RSC), brain areas located over the posterior region form a main cluster in resting state networks, partial interruption and apparent connectivity between them is indicative of prodromal AD [112,113,114]. This implies that these particular brain areas, disconnected from the rest of the brain in prodromal AD, are likely interrupted due to high metabolic activity and amyloid plaque aggregation in these particular regions [115,116,117]. Furthermore, findings in preclinical stages, such as MCI, are conflicting in brain network studies, since a few of them have found no significant disruptions of network properties (e.g., “small-world”), whereas others have found decreased FC [118,119]. Accordingly, we did not find a statistically significant difference in global brain network properties (clustering coefficient, strength and betweenness centrality) between HC and SCD nor between SCD and the rest of the groups (MCI and AD), which could indicate that brain changes in the SCD participants are too subtle to be detected in the whole brain, while at a local level we can detect network disruptions. Our results are in line with other approaches which have not found any significant differences in participants with regards to global network metrics [31,37,47,120]. Hence, it is still uncertain whether people at preclinical stages of AD would exhibit a disrupted property in their whole brain identical to that of AD individuals, and more research is necessary to cast more light on this problem.

In addition to clustering coefficient and strength, we explored the potential of betweenness centrality as a metric to differentiate the four groups. If the presence of cortical pathology in AD and its early preclinical stages suggests that a longer route for the information transfer between brain regions must take place, the global BC will have increased values. Thus, we found an escalated increase in BC among the patient groups compared to HC in parietal areas, whilst a reduction in BC was observed at a global level in more advanced stages. These results show that the disrupted interactions in advanced stages, compared to preclinical and HC, suggested that less direct paths were taken for the information transfer, passing through several cortical nodes, reducing BC in several brain areas, while at the same time increasing betweenness centrality in certain brain posterior regions. Similarly, several studies have shown that BC is lower in specific brain areas in MCI and AD with respect to HC [31,38,121]. On the other hand, decreased long-distance connectivity, indicating lower BC values of the frontal and posterior brain regions, has been found in AD compared to HC [122], suggesting that the increase in these areas lies in the fact that a compensatory mechanism is responsible for the reduced centrality in precuneus, which is a key region in the resting state networks commonly affected in AD [38]. However, we did not observe a statistically significant difference with regard to BC between the four groups, except SCD and MCI, which could be explained because SCD constitutes an early preclinical stage and the interruption is due to a compensatory mechanism or due to the small sample. Our results are in line with similar approaches that have investigated BC derived from undirected weighted networks using PCC and found no significant difference between AD and HC or other patient groups [123,124].

Despite the fact that brain disruption and brain connectome disconnection was found in SCD in the majority of the studies, small-world networks were not interrupted in the SCD group compared to HC [63]. This could also partially explain our results regarding the preserved global network metrics in this population, which suggests that no extensive disruption occurred in the brain in order to have characteristics similar to a randomized network. Although SCD preserves some network properties and several brain regions remain intact, there are extensive disruptions of local network properties indicative of those in MCI but to a lower extent [63]. Thus, SCD, compared to HC, exhibits relatively stable connections as far as network properties in a global network are concerned, hence, it has lower connections between particular brain regions over posterior brain structures. Compatible with the findings of the abovementioned studies, our results reinforce that the disrupted strength of posterior areas (parietal channels) is widely observed in SCD in a similar way to more advanced stages (e.g., MCI, AD) compared to HC. This particular “localized disconnection” has also been suggested in similar research approaches, showing that connectivity over a posterior DMN undergoes extensive disconnection with apparent connections across the AD spectrum [64]. Similarly, our results are in line with previous reported studies underlying the importance of interregional connections between frontal and parietal brain areas for episodic memory [124,125], whereas an interrupted connectivity among regions of frontal (e.g., PFC) and posterior areas (e.g., PCC) is indicative of AD [29,30,35,126,127,128]. In detail, we showed that hub regions of the DMN, such as the PCC and precuneus, presented lower activity in MCI and AD compared to HC and SCD, while reduced activity in frontal areas, such as mPFC, in the case of betweenness centrality and strength network metrics, is widely observed in people with AD compared to HC, indicating less straightforward anatomical links of contralateral brain areas [17,31]. Therefore, lower activation over the vmPFC and PCC in SCD indicates that, in contrast to AD, the hub regions of the DMN and the fronto-parietal connection are widely preserved, although to a lower extent compared to HC, which underlines that these regions work in coherence. Additionally, differences were observed among groups in the case of the cerebellum, where lower activation was detected in later stages of the disease. In line with our results, recent studies have highlighted the role of cerebellum in the AD hypothesis and its pivotal role in cognitive impairment [129,130]. Our findings support and underline the importance of a reasonable and consistent interchange among particular brain areas and support that interruption or any disconnection in these specific regions may be linked to the future development of AD.

Additionally, a recent EEG study showed that the network properties, such as increased path length, showed statistically significant differences between the HC and MCI [35]. In this common line, other studies have found that the MCI group had less network efficiency [131,132] and reduced small worldness [133] compared to the HC. The correlation between neuropsychological tests and network properties underlined that the increased cognitive impairment of the individuals’ cognitive states is associated with increased disconnection and reduced network organization. Thus, in both of the above mentioned studies, as well as in ours, the network-derived metrics based on EEGs were found to be correlated with neuropsychological tests, especially the BC, indicating that these metrics could be potentially implemented to assess the cognitive function of people at preclinical stages of AD and suggest a new diagnostic tool for both SCD and MCI. Finally, in our study, we employed the ROC curve analysis so as to define the cut-off scores and the sensitivity and specificity of each metric (clustering coefficient, strength and betweenness centrality at global and local levels). Based on our findings, the local clustering coefficient and local strength may be examined as potential markers for the detection of SCD, categorizing SCD from HC with 75% sensitivity and 64% specificity (AUC = 71%, in ROC curves), MCI from HC with 80% sensitivity and 64% specificity (AUC = 73% and AUC = 79%, respectively, in ROC curves), and AD from HC with 65% sensitivity and 82% specificity (AUC = 79% in ROC curves). An important finding deriving from the present study is that SCD individuals present network values intermediate to HC and MCI, underlying a common disconnection pattern of the brain connectome in SCD but not to the same extent as in MCI. In conclusion, our findings proved once more that AD is a “disconnection syndrome”, according to the literature to date and indicated that the resting state network was partially interrupted as cognitive impairment progresses, highlighting the importance of the early detection of cognitive impairment.

## 5. Limitations

The results of the present study should be cautiously interpreted because of some limitations. One limitation of our study is that age matching was not possible across all groups, since cognitive disturbances manifest in early 60 s but the development of AD occurs, in most cases, in later stages. Therefore, AD and HC showed statistically significant differences with respect to age. However, all four groups met the inclusion criterion of being over 60 years of age, while HC were age matched with preclinical stages of the AD continuum (SCD and MCI), showing no statistically significant differences between groups with respect to age. Another limitation is that more females than males participated in the present study. However, it is widely known that AD affects more female than male participants, which is indicative of the prevalence of AD [105,106,107], while other similar approaches have found no differences with respect to resting state activity between the gender groups [134,135]. Finally, another limitation of the current study is the lack of follow-ups in order to investigate the future progression and network changes of SCD. However, this study constitutes a novel cross-sectional study of several study groups in order to seek for potential differences in the grounds of the brain connectome among HC and people on the AD continuum. In order to overcome the above limitations, we intend, in future research, to use larger samples with several follow-ups, allowing us to examine the progression of disease. The creation of such a dataset with an adequate number of follow-ups will give us the ability to study the neuropsychological progression of the disease, and to construct efficient AD-related predictive models. Another limitation of our study is that we performed the analysis with static brain networks instead of using a more dynamic method and we adopted a bivariate connectivity estimator, which might cause issues in dense EEG networks compared to multivariate. However, since the brain connectivity analysis depends on two basic factors: (i) the measure that describes the connectivity between brain regions/electrodes, and (ii) how these connections are represented and analyzed under a unified theory and framework [136], in our analysis, we used the PCC to describe connections between brain areas under the framework of graph theory. Clearly, the choice of measure and the properties of the underlying graph affect the overall analysis. In our case, this means that, due to the correlation measure and the static nature of our brain-related graphs, we analyzed static brain networks (graphs that do not evolved with time). Another issue is that we used a bivariate connectivity estimator. However, we must point out that some brain connectivity measures may seem to be multivariate (one-to-all or all-to-all connectivity) but are in fact mass bivariate measures [137], meanwhile, the choice of a connectivity estimator that would take into account the volume conduction effect would definitely improve our methodology [136]. Furthermore, in our analysis, we used fully weighted networks since our basic goal was to study the statistical significance of connections in different brain areas, while in the future, we intend to use a thresholding approach for more specialized graph theory-based analyses [136]. Finally, we used broadband EEG activity (from 0.3 Hz to 75 Hz) since our goal was to study the effects of AD over all brain activity, while future studies would benefit from exploring the brain connectivity in specific bands (e.g., alpha rhythm), which are highly associated with connectome interruptions across the AD spectrum.

## 6. Conclusions

This is the first ever reported study which investigated brain connectivity by using HD-EEG in order to explore network changes in SCD with regards to HC, MCI and AD individuals. Therefore, our study provides pieces of evidence that SCD may actually indicate a transitional preclinical stage of AD with network changes and brain connectome interruptions. More specifically, the estimation of the clustering coefficient, betweenness centrality and strength of correlation networks restricted to parietal areas could serve as a possible biological predictor of future cognitive impairment connected to AD. However, more longitudinal research is required to extend and further investigate the underlying neurophysiological mechanisms that are associated with these particular brain network interruptions commonly occurring in SCD.

## Figures and Tables

**Table 1:** The table presents mean ± SD (standard deviation) of demographic characteristics among participants (HC = 22, SCD = 20, MCI = 30, AD = 20).

Groups				
	HC	SCD	MCI	AD
Age	67.22 (4.03)	64.90 (7.92)	70.40 (5.96)	73.20 (8.17)
Gender (M:F)	8:14	7:13	8:22	8:12
Years of Education	13.16 (4.59)	13.75 (3.29)	11.45 (4.06)	9.77 (5.51)

**Table 2:** The table shows the mean ± SD (standard deviation) of neuropsychological assessments of the participants (size in groups: HC = 22, SCD = 20, MCI = 30, AD =20). The last column of the table shows the *p*-values of one-way ANOVA.

Diagnosis	HC		SCD		MCI		AD		
Neuropsychological Tests	Mean	SD	Mean	SD	Mean	SD	Mean	SD	*p*-Value
MMSE	29.13	0.99	29.25	1.06	27.13 **	2.55	22.30	3.35	0.001
NPI	0.00	0.00	0.30	0.73	2.81	6.08	2.75	4.23	0.092
FRSSD total score	1.58 *	2.50	3.20	1.57	4.00 **	1.51	6.75	6.60	0.002
FUCAS total score	42.00	0.00	42.55	1.27	44.77 **	3.40	50.375	8.99	0.001
TRAIL-B	143.00	54.86	144.75	49.64	262.42 **	137.61	147.00	149.18	0.002
RBMT immediate recall	17.40	2.70	14.18	3.28	12.71 **	4.04	10.30	2.48	0.015
RBMT delayed recall	15.40	2.07	13.09	3.23	12.04	4.07	9.50	3.31	0.070
ROCFT copy	33.50	2.12	33.68	1.65	30.23	5.05	22.80	13.43	0.005
ROCFT delayed recall	31.00 *	1.41	22.08	5.69	13.54 **	5.76	9.90	9.16	0.001
RAVLT 1	7.33	3.05	7.23	2.75	5.19	2.08	4.60	2.07	0.035
RAVLT 2	5.00 *	0	7.35	3.83	5.33	2.19	5.40	3.84	0.201
RAVLT total score	53.33	13.86	53.88	12.56	33.38	16.09	34.00	16.85	0.001
RAVLT 4	−1.33	0.57	1.23	6.20	−1.76	2.99	−2.20	4.6	0.208
FAS	14.3	3.20	12.18	3.69	9.49 **	3.75	10.66	3.67	0.073

* HC vs. SCD—*p*-value < 0.05, ** HC vs. MCI—*p*-value < 0.01.

**Table 3:** Mean ± SD of network properties at a local level (parietal electrodes) and global level (all 256 electrodes) of the participants (HC = 22, SCD = 20, MCI = 30, AD = 20). The last column of the table shows the *p*-values of one-way ANOVA. Superscripts indicate the statistical significance between groups after independent sample *t*-tests.

		HC		SCD		MCI		AD		
		Mean	SD	Mean	SD	Mean	SD	Mean	SD	*p*-Value
Local	Clustering Coefficient	0.79	0.07	0.73 *	0.09	0.72 **	0.09	0.68 ***	0.11	0.004
	Strength	22.56	1.65	21.11 *	2.10	20.83 **	2.25	20.12 ***	2.67	0.004
	Betweenness Centrality	0.044	0.03	0.056 *	0.03	0.047 ^+^	0.02	0.06	0.02	0.431
Global	Clustering Coefficient	0.311	0.079	0.308	0.088	0.291	0.072	0.285	0.091	0.681
	Strength	99.24	18.08	97.70	20.19	94.01	16.20	91.88	21.92	0.569
	Betweenness Centrality	0.33	0.05	0.30	0.11	0.33	0.06	0.32	0.08	0.531

* HC vs. SCD—*p*-value < 0.05, ** HC vs. MCI—*p*-value < 0.01, *** HC vs. AD—*p*-value < 0.001, ^+^ SCD vs. MCI—*p*-value < 0.05.

**Table 4:** Sensitivity and specificity of clustering coefficient, strength and betweenness centrality at global and local levels for each group compared with one of the other three groups.

Groups	Global/Local	Network Property	AUC (%)	Threshold Value	Sensitivity (%)	Specificity (%)
HC vs. SCD, MCI and AD	Local	Clustering Coefficient	74	0.78	64	78
		Strength	74	22.38	64	79
		Betweenness Centrality	40	0.31	64	39
	Global	Clustering Coefficient	55	0.5	41	78
		Strength	56	106.65	41	75
		Betweenness Centrality	55	0.04	73	24
SCD vs. HC, MCI and AD	Local	Clustering Coefficient	51	0.79	90	25
		Strength	52	22.85	90	25
		Betweenness Centrality	51	0.69	80	26
	Global	Clustering Coefficient	55	0.28	70	44
		Strength	55	97.11	60	58
		Betweenness Centrality	43	0.28	75	25
MCI vs. SCD, HC and AD	Local	Clustering Coefficient	57	0.75	67	52
		Strength	57	22.14	77	40
		Betweenness Centrality	54	0.05	61	43
	Global	Clustering Coefficient	54	0.30	61	57
		Strength	54	94.48	57	58
		Betweenness Centrality	49	0.37	71	40
AD vs. HC, SCD and MCI	Local	Clustering Coefficient	66	0.70	55	75
		Strength	65	20.37	55	76
		Betweenness Centrality	66	0.05	68	62
	Global	Clustering Coefficient	55	0.32	75	41
		Strength	56	72.31	25	93
		Betweenness Centrality	51	0.28	73	27

**Table 5:** Sensitivity and specificity of clustering coefficient, strength and betweenness centrality at global and local levels for all different combinations of one to one comparisons.

Groups	Global/Local	Network Property	AUC (%)	Threshold Value	Sensitivity (%)	Specificity (%)
HC vs. SCD	Local	Clustering Coefficient	71	0.78	75	64
		Strength	71	22.34	75	64
		Betweenness Centrality	41	0.033	77	30
	Global	Clustering Coefficient	49	0.185	15	100
		Strength	51	69.829	15	100
HC vs. MCI	Local	Clustering Coefficient	73	0.78	80	64
		Strength	79	22.31	80	64
		Betweenness Centrality	45	0.036	77	32
	Global	Clustering Coefficient	44	0.259	74	36
		Strength	43	86.773	68	36
HC vs. AD	Local	Clustering Coefficient	79	0.73	65	82
		Strength	79	21.16	65	82
		Betweenness Centrality	29	0.068	79	23
	Global	Clustering Coefficient	58	0.351	85	41
		Strength	59	107.412	85	36
SCD vs. MCI	Local	Clustering Coefficient	54	0.76	70	45
		Strength	53	19.80	27	85
		Betweenness Centrality	54	0.060	40	71
	Global	Clustering Coefficient	57	0.285	54	70
		Strength	56	93.358	54	65
SCD vs. AD	Local	Clustering Coefficient	63	0.69	50	80
		Strength	62	20.41	55	70
		Betweenness Centrality	38	0.071	25	79
	Global	Clustering Coefficient	57	0.317	75	45
		Strength	59	97.879	65	55
MCI vs. AD	Local	Clustering Coefficient	58	0.70	55	70
		Strength	58	20.50	55	70
		Betweenness Centrality	35	0.058	36	58
	Global	Clustering Coefficient	51	0.241	30	79
		Strength	52	70.799	20	96

**Table 6:** Pearson correlation between network properties and neuropsychological tests for all participants at a local level.

Domain	Neuropsychological Tests	Clustering Coefficient	Strength	Betweenness Centrality
Global Cognition	MMSE	0.158	0.141	−0.254 *
Mood	NPI	−0.082	−0.083	0.149
Memory and Executive Function	RBMT immediate recall	0.167	0.146	−0.251
	RBMT delayed recall	0.205	0.186	−0.362 *
	ROCFT copy	0.119	0.085	−0.501 **
	ROCFT recall	0.169	0.149	−0.501
Learning	RAVLT recall	0.152	0.144	−0.042
	RAVLT learning	0.018	0.014	−0.062
Daily Functionality	FUCAS total score	−0.053	−0.028	0.281 *
	FRSSD total score	−0.070	−0.053	0.244
	FRSSD sleep	−0.286 *	−0.280	0.522 **

* Correlation is significant at the 0.05 level (two-tailed), ** correlation is significant at the 0.01 level (two-tailed), no superscript indicate no statistically significant difference.
